# Comparative efficacy of different salt tolerant rhizobial inoculants in improving growth and productivity of *Vigna radiata* L. under salt stress

**DOI:** 10.1038/s41598-023-44433-8

**Published:** 2023-10-14

**Authors:** Qasim Ali, Muhammad Shabaan, Sana Ashraf, Muhammad Kamran, Usman Zulfiqar, Maqshoof Ahmad, Zahir Ahmad Zahir, Muhammad Junaid Sarwar, Rashid Iqbal, Baber Ali, M. Ajmal Ali, Mohamed S. Elshikh, Muhammad Arslan

**Affiliations:** 1 Department of Soil Science, Faculty of Agriculture and Environment, The Islamia University of Bahawalpur, Bahawalpur, 63100 Pakistan; 2 Land Resources Research Institute, National Agricultural Research Centre, Islamabad, Pakistan; 3 College of Earth and Environmental Sciences, Quaid-e-Azam Campus, University of the Punjab, Lahore, 54590 Pakistan; 4 Pakistan Council for Science and Technology, Ministry of Science and Technology, Islamabad, 44000 Pakistan; 5 Department of Agronomy, Faculty of Agriculture and Environment, The Islamia University of Bahawalpur, Bahawalpur, 63100 Pakistan; 6 Institute of Soil and Environmental Sciences, University of Agriculture, Faisalabad, 38040 Pakistan; 7 Department of Plant Sciences, Quaid-i-Azam University, Islamabad, 45320 Pakistan; 8 Department of Botany and Microbiology, College of Science, King Saud University, 11451 Riyadh, Saudi Arabia; 9 Institute of Crop Science and Resource Conservation (INRES), University of Bonn, Bonn, Germany

**Keywords:** Plant hormones, Plant physiology, Plant stress responses

## Abstract

Worldwide, salinity severely affects agricultural production of crops such as mung bean in arid and semi-arid regions. In saline conditions, various species of Rhizobium can be used to enhance nodulation and induce salinity tolerance in maize. The present study conducted a pot experiment to determine the efficiency of three rhizobial isolates under different salinity conditions, such as 1.41, 4 and 6 dS m^−1^, on mung bean growth parameters, antioxidant status and yield. Results revealed that salt stress imparted adverse effects on the growth, antioxidants, yield and nodulation of mung bean. Under high salt stress conditions, fresh weights were reduced for roots (78.24%), shoots (64.52%), pods (58.26%) and height (32.33%) as compared to un-inoculated control plants. However, an increase in proline content (46.14%) was observed in high salt stressed plants. Three Rhizobium isolates (Mg1, Mg2, and Mg3), on the other hand, mitigated the negative effects of salt stress after inoculation. However, effects of Mg3 inoculation were prominent at 6 dS m^−1^ and it enhanced the plant height (45.10%), fresh weight of shoot (58.68%), root (63.64%), pods fresh weight (34.10%), pods number per plant (92.04%), and grain nitrogen concentration (21%) than un-inoculated control. Rhizobium strains Mg1, and Mg2 expressed splendid results at 1.41 and 4 dS m^−1^ salinity stress. The growth promotion effects might be due to improvement in mineral uptake and ionic balance that minimized the inhibitory effects caused by salinity stress. Thus, inoculating with these strains may boost mung bean growth and yield under salinity stress.

## Introduction

Globally, salinity severely affects agricultural production, mainly in arid to semi-arid regions. Over 800 million ha area has been estimated to be under salt stress with an annual increase of nearly 1–2%^1^. About 33% of irrigated areas worldwide and 20% of cultivated areas are under salt stress and deteriorated. Pakistan falls under arid and semi-arid regions where shortage and irregular rainfall patterns cause sodicity and salinity in fertile lands. Out of 23.80 million ha of cultivated land in Pakistan, about 6.8 million ha is adversely affected by salt stress^2,3^. Increasing salt-stressed land concerns food security by reducing crop yields by up to 50 percent annually in salt-affected agricultural zones^4^. Pulse crops are more susceptible to salinity stress than cereals and other crops.

Pulses are leguminous crops that have Rhizobium in their root nodules which contributes to atmospheric nitrogen (N2) fixation. Pulses serve as an important part of the human diet and are nitrogen fixers to improve soil fertility. Mung bean is a commonly grown pulse in Pakistan and has two growing periods in a year due to its short life span. Seeds of mung bean have high nutritional value, such as 20–25% protein and 55–65% carbohydrates, primarily starch^5^. In Pakistan, mung bean cultivation spans across approximately 215 thousand hectares, yielding a total of 141.2 thousand tons of grains annually. However, the calculated average yield of 1.1 tons per hectare falls below that of certain other countries. For instance, China and Uzbekistan achieve higher average yields of 1.41 tons/ha and 1.92 tons/ha, respective^6,7^. Due to its semi-arid location, it is quite sensitive to salinity^8^.

Salt stress influences the metabolic activities of plants to a great extent^9,10^. Salinity stress may affect growth and physiology of the plant in different ways that ultimately reduce crop yields. Presence of increased salt concentrations around root zone imposes osmotic stress leading to ion toxicity^11–13^. Osmotic stress, in turn, interferes with water uptake, cell elongation, seed germination, lateral branching, leaf development, photosynthetic rate, nutrient uptake, and subsequent translocation towards above ground plant parts, increased supply of photosynthates to meristematic regions, and ultimately, exerts adverse impact on overall plant growth^14–16^. Toxic concentrations of Na^+^ and C^l¯^ interrupts the uptake of essential nutrients such as Ca^2+^ and K^+^ and thereby, cause nutrient imbalance in plants^17–19^. There is a dire need to find the ecofriendly methods to enhance the growth and productivity of crops under salt stressed conditions.

Several physiochemical and biological approaches can be employed to minimize salinity's impacts^20^. Among physical strategies are gypsum application, irrigation water management, and scraping of surface salts, but all are expensive and less effective techniques^21^. Nowadays, numerous salinity-tolerant Rhizobium species are well-known^22^. These species have the potential to regulate the ethylene synthesis in plants^23^ and yield improvement of leguminous crops in both arid and semi-arid regions^24^.

Because of rhizobia's environmental and economic benefits, its application may be useful to achieve sustainability in cropping systems^25^. Rhizobia improves soil fertility under salinity stress and helps reintroduce crops specifically to nitrogen-deficient areas^26,27^. A recent study found that Rhizobium inoculation in mung bean under normal conditions increased the nodulation, root and shoot length, photosynthetic activity, leaf area, plant biomass and plant height^28^. Rhizobia can either directly or indirectly promote plant growth. Many plant growth hormones, such as cytokinins, gibberellins, auxins, and abscisic acid, are synthesized directly by rhizobial isolates, and many other chemicals beneficial to plant growth, such as exopolysaccharides, siderophores, ACC-deaminase, and others, are secreted^29–33^. Rhizobia also improves plant nutrient availability by mobilizing nutrients in the soil and improving soil structure^34^. Rhizobia indirectly increase plant health by increasing plant self-defense through the induction of systemic resistance^35^ against damaging insects, infections, diseases, and viruses^36^. Rhizobia has also been shown to boost legume growth and production under salt stress conditions^37,38^. There have been numerous reports of rhizobia being used to increase the number of primary roots, root proliferation, and plant growth stimulation even when the salinity level is high^39^.

As limited work has been conducted to explore rhizobia potential in enhancing the growth of legumes and biological nitrogen fixation under salinity stress, so, current work was done to find out the efficacy of Rhizobium in growth improvement and yield enhancement of crop mung bean under saline soil.

## Materials and methods

The effect of salt-tolerant rhizobial strains on mung bean growth, nodulation, antioxidant status, and yield under salt-stressed conditions was tested in a pot experiment. Soil was taken from the farm area of the Institute of Soil and Environmental Sciences (ISES), University of Agriculture in Faisalabad (UAF). Before filling the soil in the pots, it was dried, sieved, and analyzed for its physicochemical properties (Table 1).

**Table 1:** Physicochemical characteristics of soil used in the experiment.

Characteristics	Units	Value
Sand	%	50.9
Silt	%	27.8
Clay	%	21.3
Textural class		Sandy clay loam
Saturation percentage	%	33.5
pH_s_		7.8
EC_e_	dS m^−1^	1.46
CEC	Cmol_c_ kg^−1^	5.10
Organic matter	%	0.72
Total nitrogen	%	0.06
Available phosphorus	mg kg^−1^	7.34
Extractable potassium	mg kg^−1^	131

### Determination of soil texture, pH and electrical conductivity (ECe)

Determination of soil texture was made by using the method of Moodie et al.^40^. One hundred and fifty milliliters of distilled water along with forty milliliters of 1% sodium hexametaphosphate solution were added in a 400 g soil sample. The mixture was placed overnight. The soil was stirred using a mechanical stirrer, followed by recording the readings with the help of a Bouyoucos hydrometer after the soil stirring with a mechanical stirrer. In addition, a soil textural triangle was used for assessing the textural class.

Soil pH was determined from the saturated paste. For this, 250 g of soil was used, and saturated paste was prepared using distilled water. After staying for one hour, its pH was determined through a pH meter. The soil paste was extracted using a vacuum pump to determine ECe. The electrical conductivity of the extract was recorded with a digital Jenway conductivity meter.

### Soil organic matter

The organic matter content of soil was determined using the method outlined by Moodie et al.^40^ One gram of soil was well mixed with ten milliliters of 1N potassium dichromate solution and twenty milliliters of concentrated sulfuric acid. The excess was titrated against 0.1N potassium permanganate to pink end point using 150 mL of distilled water and 25 mL of 0.5 N ferrous sulphate solution.

### Determination of soil nitrogen, phosphorus and potassium (NPK)

Soil samples in triplicate were digested using Ginning and Hibbard's H_2_SO_4_ technique to estimate the N. Digestion was followed by distillation using macro Kjeldhal’s apparatus (UDK-126D, Velp-Scientifica, Italy). While Watanabe and Olsen^41^ method was used for determining available P with the help of spectrophotometer (e-300, Thermo Electron Corporation, USA). Extractable K was detected using a flame photometer (Model FP-410, Sherwood, UK) following Ryan et al.^42^ method.

### Selection of Rhizobium strains and preparation of inocula

Pre-isolated and pre-characterized three rhizobium isolates (Mg1, Mg2, and Mg3) were obtained from the Soil Microbiology and Biochemistry Laboratory, ISES, UAF. To prepare fresh inocula of all the rhizobial isolates, yeast extract mannitol (YEM) broth was used in 250 mL flasks. For growing selected isolates, 100 mL of YEM broth was inoculated with respective rhizobial strains and placed in a shaking incubator (100 rpm and 72 h). Optical density (OD) was measured after the incubation period using OD-meter, and a uniform population (OD_540_ = 0.45; 10^7^–10^8^ cfu mL^−1^) was achieved by dilution prior to seed inoculation.

### Seed inoculation

The suspension was prepared for each rhizobial isolate (100 mL), and 250 g of sterilized peat was mixed in it. For inoculation purposes, mung bean seeds were coated with this inoculated peat mixed with 50 mL of sugar-sterilized solution (10%). Autoclaved sugar and broth solution were used for the control treatment.

### Pot trial

The pot experiment was carried out in the wirehouse of the ISES, UAF. Each pot was filled with 12 kg of dried and sieved soil. There were three different levels of salinity i.e., original (1.41), 4 and 6 dS m^−1^. The measured quantity of NaCl salt was added to each pot, and after that, salt was properly mixed in order to achieve the desired level of salinity. In each pot, ten mung bean seeds that had been infected were planted. First treatment served as control (1.41 dS m^−1^) whereas, second, third and fourth treatments involved rhizobial strains (Mg1, Mg2, and Mg3) without salt stress. Similarly, fifth and sixth treatments comprised of two different salinity levels, 4 and 6 dS m^−1^ without any inoculation. While in the subsequent treatments, these rhizobial strains were tested individually against different salinity levels (1.41, 4 and 6 levels dS m^−1^). Each treatment had six replications. Pots were arranged in the wirehouse in accordance with a completely randomized design (CRD) at the ambient light and temperature. In each pot, the recommended amount of nitrogen, phosphorus, and potassium fertilizers (20: 60: 60 kg ha^−1^), which were applied as urea, diammonium phosphate, and sulphate of potash, respectively, were used. All fertilizers were applied as basal dose at the time of sowing. Pots were irrigated using good quality canal water. Thinning was done after almost fifteen days of germination. At the flowering stage, plants from three replicates were uprooted to evaluate the contribution of different strains to nodulation. After a period of sixty days, triplicate fresh leaf samples were obtained and examined to determine the relative water content, as well as the proline, salt, and potassium levels in the leaves. When the plants had reached their full maturity, three replicates were harvested, and data regarding growth and yield metrics were collected. Besides, chemical analysis was used to assess the amounts of nitrogen (N), phosphorus (P), potassium (K^+^), and sodium (Na^+^) present in various plant samples.

### Determination of relative water content (RWC)

The formula given by Gonzalez and Gonzalez-Vilar^43^ was used to determine RWC:

$${\text{RWC }} = {\text{ FW }}{-}{\text{ DW}}/{\text{FTW }}{-}{\text{ DW}}$$

### Determination of proline content in plant samples

Bates et al.^44^ method was used to determine free proline contents. A leaf sample of one gram was homogenized in sulfosalicylic acid at a concentration of 3 percent before being filtered using Whatman filter paper No. 2. Following the addition of glacial acetic acid and acid ninhydrin, the mixture was placed in a water bath and heated to a temperature of 100 °C for 1 h. After that, the reaction was stopped by placing the mixing in an ice bath. The mixture was extracted with toluene, and its absorbance at 520 nm was determined. The amount of proline was calculated using a standard curve and given as mol g^−1^.

### Antioxidant defense

Antioxidant enzymes, including catalase (CAT) and ascorbate peroxidase (APX), were measured in leaves using spectrophotometry. The activity of the CAT enzyme was assessed using the method of Aebi^45^. Furthermore, APX activity was measured by following the method of Nakano and Asada^46^. In addition, superoxide dismutase (SOD) activity was measured using nitro blue tetrazolium (NBT) method^47^ and was based on the photoreduction of NBT. Malondialdehyde (MDA) contents were measured using Heath and Packer's^48^ approach for the thio-barbituric Acid (TBA) reaction.

### Determination of NPK in leaves

Following harvest, 0.1 g of oven dried and crushed plant sample in triplicate was digested using Wolf's Method^49^. Nitrogen was measured using Kjeldhal's method^50^; potassium was measured with a flame photometer (Mason, 1963); and phosphorus was measured with a spectrophotometer using the standard protocols^51^.

### Statistical analysis

The recorded data were analyzed statistically by two-way analysis of variance technique (ANOVA) using a computer-based statistical software Statistix 8.1 (Statistix, USA)^52^, and the difference between the data means was determined by using Duncan’s Multiple Range post-hoc test (p ≤ 0.05)^53^. Means and standard errors were calculated with the help of Microsoft Excel.

### Plant guidelines

All the plant experiments were performed by relevant institutional, national, and international guidelines and legislations.

## Results

Salinity stress negatively impacted mung bean growth, antioxidant defense, yield and ionic parameters under salinity stress in pots. Rhizobial inoculation improved all the parameters significantly at multiple salinity levels. However, inoculation caused more improvement in parameters at higher levels of salinity when compared with respective uninoculated controls.

### Growth parameters

Data showed that NaCl salinity adversely affected mung bean growth parameters more than its respective control (Table 2). At 1.41 dS m^−1^ salt level, the most remarkable increase in the shoot fresh (17.11%) as well as dry weight (20.94%), fresh weight (32.68%) and dry weight of root (46.01%) was recorded in Mg1 isolate than respective uninoculated control. However, Mg2 and Mg3 behaved differently for different parameters when compared with uninoculated control. At 4 dS m^−1^ salinity level, plant maximum height (37.20%), shoot fresh (25.48%) and dry weight (54.24%), root length (29.63%), and dry weight of root (59.28%) compared to uninoculated control was recorded in mung bean plants which were inoculated with Mg1. Similarly, at a salinity level of 6 dS m^−1^, Mg3 proved best due to the increased height of the plant (82.17%), fresh (142%) and dry weight of shoot (258%), root length (57.25%), fresh (168%) and dry weight of root (294%) than un-inoculated control. Strains Mg1 and Mg2 responded differently at different parameters. However, in the case of root fresh weight, Mg2 performed best and showed an increase of 58.17% than the respective uninoculated control.

**Table 2:** Effect of rhizobial inoculation on growth and nodulation of mung bean under saline conditions.

Treatments	Plant height (cm)			Shoot fresh weight (g)			Shoot dry weight (g)		
	Original	4 dS m^−1^	6 dS m^−1^	Original	4 dS m^−1^	6 dS m^−1^	Original	4 dS m^−1^	6 dS m^−1^
Control	31.23d–f	30.17ef	21.13g	35.06a–c	30.53c	12.44e	6.16c	4.72e	1.48g
Mg1	41.17ab	41.83a	32.57c–e	41.06a	38.31ab	18.65de	7.45a	7.28ab	3.32f
Mg2	37.90a–d	39.90ab	25.57fg	36.42a–c	33.75bc	20.41d	6.35c	5.95cd	2.73f
Mg3	36.67a–d	35.00b–e	38.50a–c	39.71ab	35.69a–c	30.11c	7.43a	6.53bc	5.31de

Treatments	Root length (cm)			Root fresh weight (g)			Root dry weight (g)		
	Original	4 dS m^−1^	6 dS m^−1^	Original	4 dS m^−1^	6 dS m^−1^	Original	4 dS m^−1^	6 dS m^−1^
Control	33.57de	31.5e	23f	8.72b	4.47ef	1.90g	2.13c	1.67d	0.59e
Mg 1	37.2bcd	40.83b	37.33bcd	11.57a	6.04d	3.63f	3.11a	2.66ab	1.46d
Mg 2	31.5e	36.37cd	35.67cd	9.55b	4.75e	3.84f	2.51bc	2.17c	1.37d
Mg 3	46.87a	37.9bc	36.17cd	9.47b	7.07c	5.19e	2.28bc	2.26bc	2.33bc

Treatments	Number of nodules			Nodule fresh weight (g)			Nodule dry weight (g)		
	Original	4 dS m^−1^	6 dS m^−1^	Original	4 dS m^−1^	6 dS m^−1^	Original	4 dS m^−1^	6 dS m^−1^
Control	2.33fg	–	–	–	–	–	–	–	–
Mg 1	17.33b	9.00cd	6.00de	0.14c	0.13cd	0.05f	0.04c	0.01d	0.007f
Mg 2	27.33a	9.67c	4.67ef	0.24b	0.09de	0.05ef	0.07b	0.01d	0.009e
Mg 3	29.33a	7.33c–e	–	0.31a	0.12de	–	0.09a	0.04c	–

According to Duncan’s multiple range test (*p* < 0.05), data means having the same letter(s) are statistically at par.

### Nodulation

Adverse effects of salt stress on the number of nodules and their fresh weights were observed, pronounced with elevation in salt concentration (Table 3). Negative impacts of salt stress on nodule number and their fresh weight were recorded, pronounced with the elevation in salt concentration (Table 3). In non-saline control, nodules had the highest number due to Mg3 isolate inoculation, which was 69.33% higher than the nodules obtained due to the inoculation of Mg1 isolate. Mg1 increased 6.42 folds compared to the respective uninoculated unstressed plants at par with isolate Mg2. Likewise, at 4 dS m^−1^, the highest nodules number (ten nodules/plant) were observed in Mg3 inoculated plants, with a significant increase of 32% over the plants inoculated with Mg3 isolate. The remaining strains (Mg3 and Mg1) produced 7 and 9 nodules per plant, respectively. However, salt stress inhibited nodulation in control plants. At the highest level of salts, i.e., 6 dS m^−1^, uninoculated plants failed to develop nodules again. While the plants inoculated with Mg1 produced 50% more nodules than the Mg2-inoculated plants.

**Table 3:** Effect of rhizobial inoculation on yield parameters and nutrient concentrations of mung bean under saline conditions.

Treatments	No. of pods plant^−1^			Pod fresh weight (g)			Grain yield plant^−1^ (g)		
	Original	4 dS m^−1^	6 dS m^−1^	Original	4 dS m^−1^	6 dS m^−1^	Original	4 dS m^−1^	6 dS m^−1^
Control	7.33bc	6.33cd	3.33e	5.53cd	6.42bc	2.31f	1.98de	1.77e	–
Mg1	9.67ab	6.00cd	5.33d	8.39a	6.42bc	6.32bc	2.91a	1.95de	1.26fg
Mg2	10.00a	8.33ab	6.67b–d	7.23ab	6.49bc	3.94de	2.17cd	2.46bc	1.16g
Mg3	8.33ab	6.67b–d	7.67bc	7.06a–c	6.99a–c	4.07de	2.79ab	2.27cd	1.60ef

Treatments	N (%) in grains			P (%) in grain			K (%) in leaves		
	Original	4 dS m^−1^	6 dS m^−1^	Original	4 dS m^−1^	6 dS m^−1^	Original	4 dS m^−1^	6 dS m^−1^
Control	1.72d	1.40e	1.19e	0.22e	0.12d	0.15c	1.75b–d	1.54d	1.33e
Mg1	2.15c	1.74d	1.81d	0.29d	0.26c	0.23d	2.26a	1.91b	1.67d
Mg2	2.76a	2.43b	1.85d	0.3d	0.28a	0.2b	2.25a	2.24a	1.67cd
Mg3	2.58ab	2.43e	1.41e	0.29e	0.23ab	0.2b	2.32a	1.85bc	1.65d

Treatments	Na (%) in leaves			K/Na ratio in leaves			Protein (%) in grains		
	Original	4 dS m^−1^	6 dS m^−1^	Original	4 dS m^−1^	6 dS m^−1^	Original	4 dS m^−1^	6 dS m^−1^
Control	0.68c	0.81b	0.95a	2.58e	1.96f	1.42g	10.73d	8.77c	7.42e
Mg1	0.60d	0.53f	0.62d	3.77bc	3.60cd	2.70e	13.46c	10.85d	11.31d
Mg2	0.59f	0.56ef	0.62d	3.82bc	4.09ab	2.75e	17.28a	15.21b	11.56d
Mg3	0.55f	0.56ef	0.47g	4.22a	3.34d	3.50cd	16.12ab	15.18b	8.78e

According to Duncan’s multiple range test (*p* < 0.05), data means having the same letter(s) are statistically at par.

Similarly, at original (1.41 dS m^−1^) salinity, in the case of nodule fresh weight, the highest nodule fresh weight was noted due to the strain Mg3, which was 122% more than the respective uninoculated control followed by Mg2 isolate that statistically enhanced the fresh weight of nodule per plant by 98.8% than uninoculated control. At 4 dS m^−1^ salinity level (medium), uninoculated control plants could not develop nodules. While Mg1 isolate develop nodules with maximum fresh weight (0.13 g) followed by Mg3 (0.12 g). At 6 dS m^−1^ salinity level (highest), Mg2 was the best isolate regarding the nodule fresh weight per plant (0.54 g), followed by Mg1 with 0.053 g per plant nodules fresh weight.

### Yield parameters

Data exhibited the effect of inoculation with rhizobia on pod number per plant, pod fresh weight and grain yield per mung bean plant under stressed salt conditions (Table 3). At low salinity (1.41 dS m^−1^), Mg2, Mg1 and Mg3 inoculation significantly increased pods number per plant by 36.43, 31.92 and 13.64%, respectively, compared to uninoculated control. On the other hand, at 4 dS m^−1^ (medium salinity level), Mg2 and Mg3 strains caused 31.60 and 5.31% increase in the number of pods per plant, respectively, than uninoculated control. However, at 6 dS m^−1^, an increase in pod number/plant by rhizobial strains Mg3, Mg2 and Mg1 strains was estimated to be increased by 130, 100 and 60%, respectively, than respective uninoculated control. While in the case of pods' fresh weight, a maximum increase was noted for Mg1 (173%) at 6 dS m^−1^, followed by the same strain (51.72%) at the original (1.41 dS m^−1^) salinity level than the respective uninoculated control. However, a non-significant increase in pods' fresh weight was noted at 4 dS m^−1^ due to rhizobial inoculation. While in the case of grain yield, inoculation with Mg1, Mg3 and Mg2 resulted in 46.97, 40.91 and 9.6% increase, respectively, with respect to respective uninoculated control, whereas Mg2 strain caused a maximum increase (38.98% than respective uninoculated control) in yield of mung bean grain at 4 dS m^−1^. At 6 dS m^−1^ (highest salinity level), uninoculated control plants could not develop grains. While Mg3 inoculated plants produced the maximum amount of grains (1.60 g plant^−1^), followed by plants inoculated with Mg1 isolate (1.26 g plant^−1^), which was 27.24% lower than the produce of Mg3 isolate and 8.93% higher than the produce of Mg2 isolate.

### Ionic contents

Data showed the accumulation of ionic and protein contents of mung bean under salt-stressed conditions (Table 3). An increase in salt concentration significantly decreased the grain concentration of nitrogen and phosphorous, leaves potassium concentration, K^+^/Na^+^ ratio in leaves, and grains protein content of uninoculated plants. However, the Na concentration of leaves of uninoculated plants increased with the increase in salt concentration. The maximum increase in the concentration of nitrogen (60.47%), phosphorus (36.36%) and protein (60.47%) in grain than respective uninoculated control was recorded due to Mg2 strain at original (1.41 dS m^−1^) salinity. In comparison, Mg3 caused a maximum increase in potassium concentration (32.57%) and K^+^/Na^+^ ratio (63.57%) in leaves as compared to respective uninoculated control at this same salinity level. At 4 dS m^−1^, the greatest increase in the concentration of nitrogen, phosphorus, protein in grains, and potassium and K^+^/Na^+^ ratio in leaves was recorded due to Mg2 strain, which was 73.57, 133.3, 73.57, 45.45 and 110.5% than uninoculated control, respectively. Almost similar results trend was observed by Mg2 strain at 6 dS m^−1^ (highest salinity level). In the case of sodium concentration in leaves of mung bean plant, Mg isolates were efficient in reducing the accumulation of Na^+^ in plants which was 20% lower than respective uninoculated plants at the original (1.41 dS m^−1^) salinity level, i.e. 1.41 dS m^−1^ and statistically similar to the rest of the treatments. At 4 dS m^−1^, Mg3 and Mg2 gave similar results and reduced Na^+^ accumulation by 35% each when compared with the respective uninoculated control. Both these treatments were similar statistically to Mg1 but different from the respective uninoculated control. At 6 dS m^−1^, Mg3 isolate caused a 50% decrease in the accumulation of Na^+^ concentration of leaves than uninoculated control plants, followed by Mg1 and Mg2 that caused a concentration of 35% each over a respective uninoculated control.

### Relative water content (RWC)

**Figure 1:**
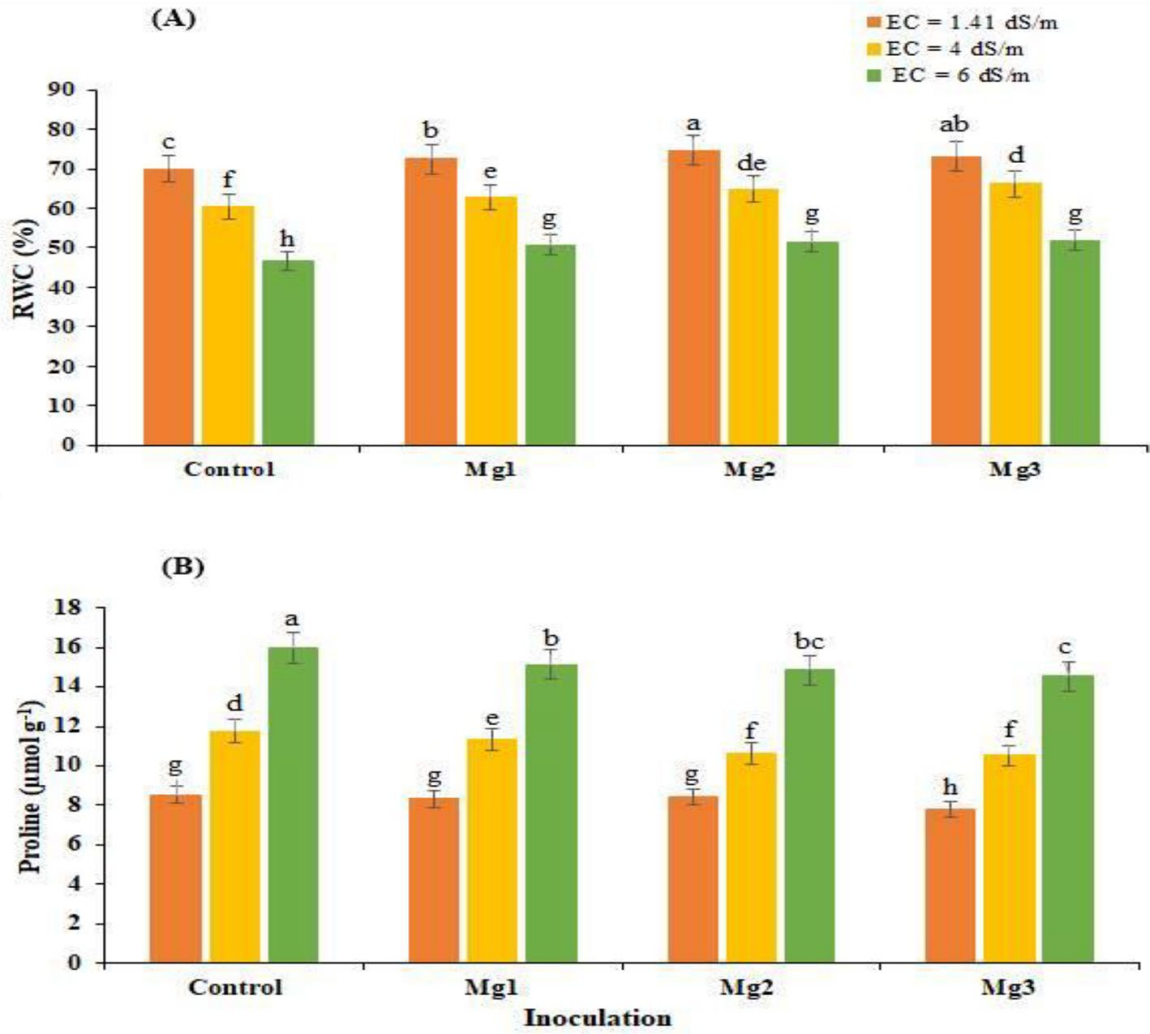
Effect of *Rhizobium* inoculation on (**A**) relative water content (RWC; %) and (**B**) proline content of mung bean under salt-stressed conditions (average of three replicates). *Mg1* Rhizobial strain 1, *Mg2* Rhizobial strain 2, *Mg3* Rhizobial strain 3.

Data concerning the impact of rhizobial inoculation on the relative water contents of mung bean plants indicated a tremendous increase in the RWC at all the applied salinity levels (Fig. 1A). A maximum increment of 6.76% in the RWC at lower salinity levels, such as 1.41 dS m^−1^, was pragmatic by inoculation of Mg2 compared to control. However, inoculation of Mg3 and Mg1 enhanced RWC by 4.47 and 3.52%, respectively, than respective uninoculated control. Relative water contents (RWC) were also increased by 9.65% at a medium salinity level of 4 dS m^−1^ with the inoculation of Mg3 compared to the uninoculated control treatment. Similarly, at medium salinity level, Mg2 and Mg1 uplifted the RWC by 7.28 and 4.13%, respectively, than the control. Similarly, Mg3 provided tremendous results than other treatments at high salinity levels (6 dS m^−1^) and significantly improved the RWC of mung bean by 10.82% than the uninoculated control. However, inoculation of Mg2 and Mg1 promoted RWC by 10.04 and 8.34%, respectively, at a high salinity level of 6 dS m^−1^ compared to the control treatment (Fig. 1A).

### Proline content

Reduction in proline content of inoculated plants ranging from 8.31 to 10.46% to uninoculated control was recorded due to the Mg3 isolate under saline conditions (Fig. 1B). At the original salinity level, i.e., 1.41 dS m^−1^, strain Mg3 resulted in 8.31% lower proline accumulation than uninoculated control. Mg1 and Mg2 accumulated 1.21 and 2.5% less proline contents than the respective uninoculated control. Similarly, Mg3 isolate resulted in 10.5 and 9% reductions in the proline content of mung bean plants under 4 and 6 dS m^−1^ salinity levels, respectively, followed by Mg2 isolate that caused a decrease of 7.06 and 6.9% in proline content at 4 and 6 dS m^−1^ level of salt concentration in comparison to uninoculated control respectively (Fig. 1B).

### Antioxidants

**Figure 2:**
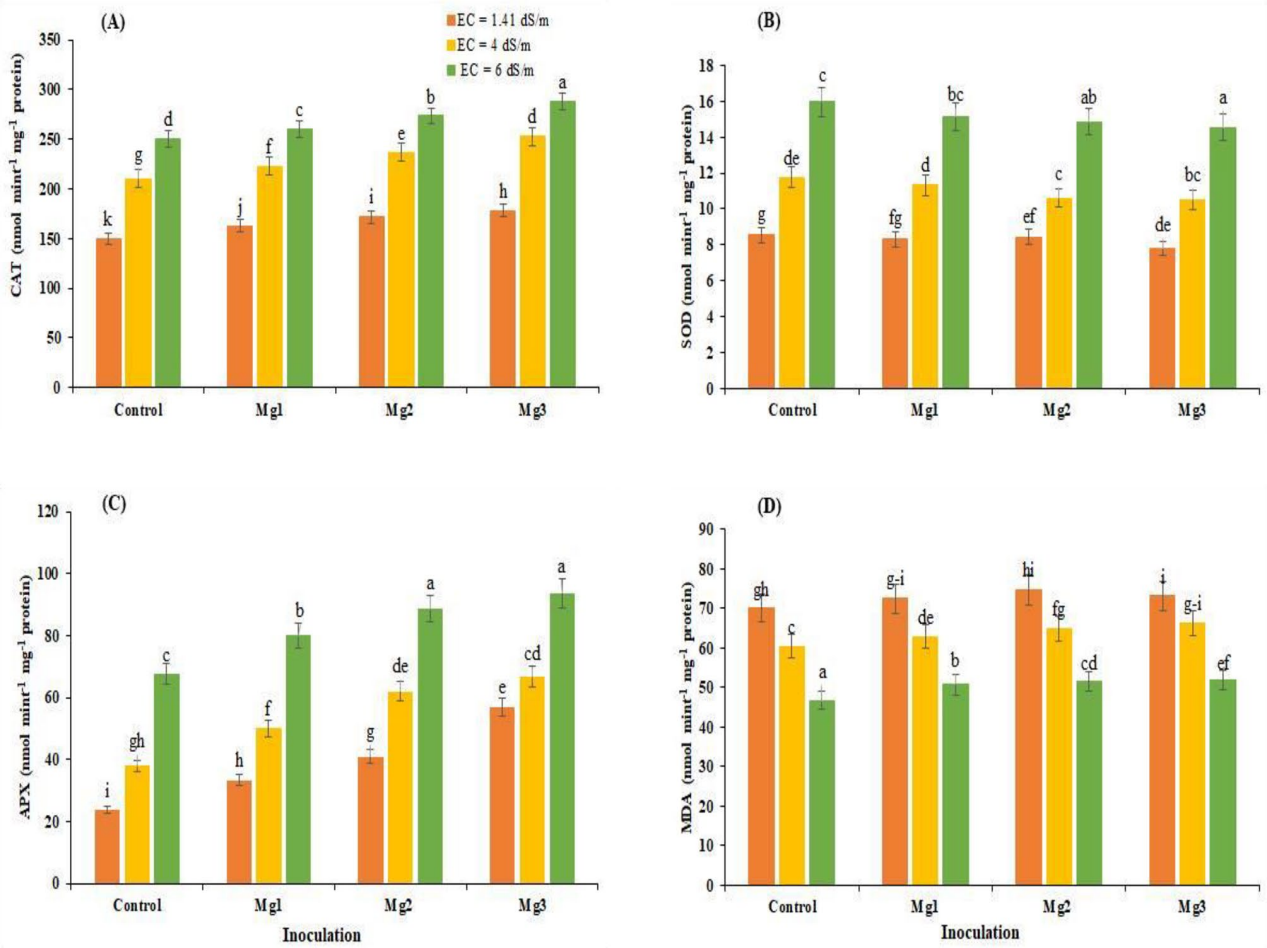
Effect of *Rhizobium* inoculation on (**A**) catalase (CAT), (**B**) superoxide dismutase (SOD), (**C**) ascorbate peroxidase (APX) activities and (**D**) malondialdehyde (MDA) contents in mung bean leaves under salt-stressed conditions (average of three replicates). *Mg1* Rhizobial strain 1, *Mg2* Rhizobial strain 2, *Mg3* Rhizobial strain 3.

Salinity stress causes oxidative stress in plants, which produce different antioxidants in their response. Regulation of other antioxidants under abiotic stresses indicates plants’ ability to tolerate oxidative stress. The findings of current study revealed that salt stress led to a substantial increment in the production of different antioxidants. However, the application of bacterial isolates (Mg1, Mg2, Mg3) boosted the levels of antioxidants such as catalase, ascorbate peroxidase (APX), and superoxide dismutase (SOD) under salt stress as compared to uninoculated control. Among different rhizobial isolates, strain ‘MG3’ caused maximum production of CAT (15, 40 and 48%) (Fig. 2A), SOD (39, 60 and 67%) (Fig. 2B), and APX (58, 64 and 74%) (Fig. 2C) and against salinity stress of 1.41, 4 and 8 dS m^−1^, respectively.

### Malondialdehyde (MDA) contents

Salt stress also triggered lipid peroxidation in mung bean by producing malondialdehyde, where maximum MDA contents were observed under 6 dS m^−1^ salt concentration. Rhizobacterial isolates reduced the MDA concentration under all salinity levels. Among different rhizobial strains, inoculation of isolate ‘Mg3’ resulted in a maximum decrease of 40, 50 and 90% against salinity stress of 1.41, 4 and 8 dS m^−1^, respectively, in MDA content (Fig. 2D).

## Discussion

Salinity stress hampers the growth and yield of crops by reducing the photosynthetic activity through reduced gaseous exchange, altering the morphological development, disrupting the membrane functions and affecting the activities of antioxidants^54–56^. In addition, it causes a reduction in the productivity of plants by increasing Na^+^/ K^+^ ratio, ionic imbalance and decreasing the N fixation, nutrient metabolism, ionic content of plant and leaf relative water content^57–59^. Nowadays, use of microorganisms has gained worldwide attention to improve leguminous crop production. There is a need to understand the mechanism behind the growth and yield promotion by Rhizobium, and all other factors that halt crop productivity under saline soil conditions. Therefore, current study evaluated different rhizobia strains for promoting mung bean growth, yield and ionic content under saline soil conditions.

Inhibition of nodule formation was observed at higher concentrations of salts which might be due to the collapse of root hair structure, leading to a decline in the growth of root hairs. Thus, it can be concluded that failure to form nodules due to higher salinity levels negatively affects the growth of plants^60^. Salinity affected nodule initiation, lowered the number, weight, and N-fixing efficiency of mungbean nodules^69^, resulting in a considerable fall in leghaemoglobin concentration, which declined with nodule ageing due to irreversible oxidation. Rhizobium inhibition of root colonization was the primary cause of inadequate nodulation^70^. Despite the presence of nodules, N fixation was largely reduced in affected plants growing at 6 dS m^−1^. High concentrations of Na^+^ and Cl^–^ present in the root and shoots of mung bean under salt stress can also disturb osmotic pressure and metabolic pathways of plants, especially in leaves because shoots can accumulate a higher amount of Cl^–^ and Na^+^ compared to roots^61,62^. Roots can sustain the tolerable limits of NaCl because of their ability to regulate the levels of NaCl through different mechanisms. In case of Na^+^ toxicity, Na^+^ replaces the K^+^ at binding sites, and disrupts cellular functions. There are more than 60 enzymes which require K^+^ ions for their activation. Na^+^ cannot substitute in this role^62,63^. Consequently, higher Na^+^ to K^+^ ratios or elevated concentrations of Na^+^ can interrupt the numerous enzymatic activities in cells. Furthermore, a high concentration of K^+^ ions is required to bind tRNA to the ribosome during translation process^64,65^. The negative effect on growth parameters and low yield of plants due to disorders in the protein synthesis process is caused by high Na^+^ concentration. These findings are confirmed by the conclusions of Hasanuzzaman et al.^66^. Similarly, Ahmad et al.^67^ examined that the production of dry mass of mung bean plants gradually decreased with an increase in NaCl level. Moreover, they observed that salinity stress adversely affected pods and nodule formation. Likewise, Panwar et al.^68^ found that salinity levels of 5 and 10 dS m^−1^ in soil caused a 17–26.8% decline in mung bean biomass, whereas seed weight was reduced by 18.6–26% at the same salinity levels. In contrast, pod number/plant and fresh weight under rhizobium inoculation increased significantly. Nyoki and Ndakidemi^77^ also found similar results while studying the effects of Rhizobium on cowpea. Similarly, Kyei-Boahen et al.^78^ observed a marked improvement in pods number plant^−1^ and seeds pod^−1^ in inoculated cowpea plants than control plants. Inoculation with rhizobial isolates improved the root length, fresh, dry weight of the shoot and root at multiple salinity levels. The study of Simon et al.^79^ revealed similar results. Ahmed et al.^80^ described a significant increment in seed-inoculated plant yield using inoculum.

Plants in a salty environment acquire proline and glutamine while increasing the content of amino di-carboxylic acid^71,72^. In the present study, improved grain yield might be due to increased dry weight, nodules number plant^−1^ and photosynthetic activities^73^. Tena et al.^74^ obtained similar results while studying the symbiotic efficiency of Rhizobium inoculation in lentils, and recorded 59% increase in grain yield of inoculated plants. Lamptey et al.^75^ also reported an improvement in soya bean seed yield due to inoculation. Rhizobial inoculation in current study enhanced the nodulation, improving the atmospheric N_2_ fixation ability. Therefore, high N utilization might cause improved growth and ultimately, plant yield. Similarly, Mondal et al.^76^ found that Rhizobium enhanced N_2_ utilization and nodule formation resulting in a high seed yield. They used various Rhizobium strains to investigate their effects on nodule formation, and mung bean yield under salinity stress. There was a significant difference among both uninoculated and inoculated plants. High proline contents were accumulated in plants due to the adoption of different stress resistance mechanisms under salinity stress^87,88^. However, Rhizobium inoculation significantly reduced proline contents by regulating the concentration of K^+^, Na^+^, P and K^+^/Na^+^ ratio in different parts of plants, which resulted in the reduction of salinity adverse effects and lower accumulation of proline content^87,88,92^.

A decrease in nitrogen concentration and protein content due to salinity stress, and improvement in these parameters through inoculation was observed in the study. Many previous scientists have witnessed the drastic effects of salinity on the N concentration and protein content of plants^81,82^. Chakrabarti and Mukherjee^83^ reported that different NaCl concentrations caused significant reductions in total N, the concentration of N present in tissues, protein and amino acids contents, N_2_ fixation and overall growth of plants. However, improvement in the N concentration of leaves and protein content due to the rhizobium inoculation under saline conditions correlates with the finding of many other scientists^84^. High N concentration of leaves and protein content might be due to increased N fixation by rhizobium strains^76^. Moreover, salinity has an impact on the acquisition of N and P by plants; it reduces their uptake through the root system while increasing Na uptake. According to our findings, plants with high salt concentrations have lower N and P contents while salt-treated plants have extremely high Na contents. Increased Na concentrations in plants have been found to reduce the accumulation of other elements like N, P, and K, causing competition in uptake, passage, or dissemination, and altering cationic and anionic ratios, such as Na^+^/K^+^ and Cl/NO_3_. By competing with K^+^ at protein-binding sites, Na^+^ damages plants by impeding the function of enzymes^85^. The interaction between Na and NH_3_ or Cl^−^ and NO_3_^−^, as well as the toxicity of certain ions like Na, S, and Cl, may be the cause of the decreased N absorption in plants under saline conditions. This, in turn, reduces the absorption and accretion of other crucial nutrients^86^. Additionally, salt stress raises the amount of Na in cell cytoplasm, which substitutes the cytosolic K, and causes Na^+^/K^+^ ratio to rise^85^.

Relative leaf water content indicates a plant’s capability to maintain water status. Therefore, it can be used as a criterion to examine salt stress effects on mung bean plants grown under salinity stress. Plants exposed to salt stress showed a decrease in relative water content. Several studies confirmed a decrease in RWC of salt-stressed plants^87,88^. Reduced relative water content of leaves in the present study might be due to the lower water uptake under higher salt concentrations^89^. It might also be due to the retarded flow of sap flux that results in reduced root hydraulic conductivity with a possibility of lower leaf RWC^90^. However, improvement in the relative water content of stressed plants due to inoculation of rhizobia has been recorded by many researchers^87,88,91^.

Antioxidant enzymes are crucial in detoxifying ROS, which are harmful and accumulate in plants under salt stress. We observed an increase in defensive antioxidant enzyme activity in inoculated plants compared to uninoculated plants under salt-stress conditions. These results are in line with Wu et al.^93^, who reported that PGPR inoculation raised SOD activity and improved resistance to salt stress in willows. Under salt stress, *E. cloacae* PM23-treated maize plants substantially enhanced the activities of APX (14–24%), SOD (23–36%) and POD (26–36%)^94^. The current study's findings support the idea that each isolate has a distinct enzymatic capability that may be enhanced in stressful and non-stressful circumstances. Antioxidant enzymes increase concurrently with a reduction in other biochemical parameters like MDA. Previous research showed that PGPR strains of Enterobacter cloacae HSNJ4 increased antioxidant systems of canola and sweet corn and Pseudomonas fluorescens against salt stress because MDA contents were reduced due to increasing antioxidant production^95–97^.

## Conclusion

Salinity stress drastically affected mung bean plants' growth, yield and ionic parameters under salinity stress. However, rhizobial inoculation significantly improved all the parameters under all the salinity levels. Different strains performed differently under varied salt concentrations. Mg3 performed excellently in enhancing the growth, yield and ionic parameters under the highest salt concentration, i.e., 6 dS m^−1^. Mg1 and Mg2 performed better at lower salt stress, i.e., 1.41 and 4 dS m^−1^. The obtained results clearly demonstrate that tested rhizobacterial strains can uplift the growth and yield of mung bean grown in marginal saline lands.

## Data Availability

All data generated or analyzed during this study are included in this published article.
